# Effect of a low-cost, behaviour-change intervention on latrine use and safe disposal of child faeces in rural Odisha, India: a cluster-randomised controlled trial

**DOI:** 10.1016/S2542-5196(21)00324-7

**Published:** 2022-02-09

**Authors:** Bethany A Caruso, Gloria D Sclar, Parimita Routray, Corey L Nagel, Fiona Majorin, Steven Sola, William J Koehne, Thomas Clasen

**Affiliations:** a Hubert Department of Global Health, Rollins School of Public Health, Emory University, Atlanta, GA, USA; b Gangarosa Department of Environmental Health, Rollins School of Public Health, Emory University, Atlanta, GA, USA; c Department of Epidemiology, Rollins School of Public Health, Emory University, Atlanta, GA, USA; d Independent Consultant, Bhubaneswar, India; e College of Nursing, and College of Public Health, University of Arkansas for Medical Sciences, Little Rock, AR, USA; f Department of Disease Control, Faculty of Infectious and Tropical Diseases, London School of Hygiene and Tropical Medicine, London, UK

## Abstract

**Background:**

Uptake of Government-promoted sanitation remains a challenge in India. We aimed to investigate a low-cost, theory-driven, behavioural intervention designed to increase latrine use and safe disposal of child faeces in India.

**Methods:**

We did a cluster-randomised controlled trial between Jan 30, 2018, and Feb 18, 2019, in 66 rural villages in Puri, Odisha, India. Villages were eligible if not adjacent to another included village and not designated by the Government to be open-defecation free. All latrine-owning households in selected villages were eligible. We assigned 33 villages to the intervention via stratified randomisation. The intervention was required to meet a limit of US$20 per household and included a folk performance, transect walk, community meeting, recognition banners, community wall painting, mothers’ meetings, household visits, and latrine repairs. Control villages received no intervention. Neither participants nor field assessors were masked to study group assignment. We estimated intervention effects on reported latrine use and safe disposal of child faeces 4 months after completion of the intervention delivery using a difference-in-differences analysis and stratified results by sex. This study is registered at ClinicalTrials.gov, NCT03274245.

**Findings:**

We enrolled 3723 households (1807 [48·5%] in the intervention group and 1916 [51·5%] in the control group). Analysis included 14 181 individuals (6921 [48·8%] in the intervention group and 7260 [51·2%] in the control group). We found an increase of 6·4 percentage points (95% CI 2·0–10·7) in latrine use and an increase of 15·2 percentage points (7·9–22·5) in safe disposal of child faeces. No adverse events were reported.

**Interpretation:**

A low-cost behavioural intervention achieved modest increases in latrine use and marked increases in safe disposal of child faeces in the short term but was unlikely to reduce exposure to faecal pathogens to a level necessary to achieve health gains.

**Funding:**

The Bill & Melinda Gates Foundation and International Initiative for Impact Evaluation.

## Introduction

A lack of safe sanitation, including inconsistent use of available facilities, is a public health problem. Open defecation and unsafe disposal of child faeces contaminate environments, enabling faecal-oral and helminth infections and insect vector diseases that might result in growth faltering, pneumonia, anaemia, impaired cognitive function, anti-microbial resistance, and death, as well as effects on wellbeing including mental health, safety, economic productivity, and school absence.^1^, ^2^

An estimated 9% of the global population practices open defecation.^3^ Sanitation coverage and use gains made in India have been substantial. Bolstered by the Swachh Bharat Mission (SBM),^4^ between 2000 and 2017, the proportion of the Indian population practicing open defecation decreased from 73% to 26% and the proportion using at least basic sanitation increased from 16% to 60%.^3^ The Government of India declared India open-defecation free on the target date (Oct 2, 2019), although the achievement has been questioned, including regarding access to and use of sanitation facilities.^5^

Latrine access enables use, but does not guarantee it. A post hoc regression analysis assessing the effect of latrine coverage on use found that every 10% increase in coverage amounted to only a 5·8% increase in use.^6^ In India, a study found substantial reductions in open defecation in rural areas between 2014, when SBM was launched, and 2018. However, the reduction was attributed primarily to the increase in latrine coverage; the proportion of latrine owners practicing open defecation did not change from 2014 to 2018 (approximately 23% both years),^7^ suggesting that SBM was not changing behaviour of latrine owners.

Many barriers to latrine use exist that extend beyond access, including perceptions of open defecation as cleaner, healthier, more convenient, or preferred, compared with latrine use; a habit;^8^, ^9^, ^10^ fear of pits filling and the need to empty them;^10^ the perception that latrines are only for women;^9^ and poor design and challenging access to water.^9^, ^11^ For environments to be free of faecal contamination and not pose risks to health, interventions are needed that are specifically designed to change defecation behaviours, particularly among those already owning latrines.^12^

**Research in context**

**Evidence before this study**

Most previous sanitation intervention trials in low-income countries have focused on improving sanitation access to improve health outcomes; two trials in rural India identified no effect on health, explained by low uptake of facilities. A 2017 systematic review and meta-analysis assessed the effect of sanitation interventions on latrine coverage and use and how sanitation structural and design characteristics are associated with use. Observational and intervention studies from Jan 1, 1950, to Dec 31, 2015, were identified from electronic databases (British Library for Development Studies; Campbell Library; ClinicalTrials.gov; Cochrane Library; Embase; EBSCO [CINHAL, PsychInfo]; LILACS; POPLINE; ProQuest; PubMed; Research for Development, Sanitary Engineering and Environmental Sciences; Social Science Research Network; Sustainability Science Abstracts; Web of Science; and International Initiative for Impact Evaluation), implementer and conference proceedings websites (Carter Center, Center for Disease Control and Prevention Global WASH, International Water Association, Menstrual Hygiene Management in WASH in Schools Virtual Conference, Stockholm Environment Institute, Stockholm World Water Week Conference, University of North Carolina Water and Health Conference, UNICEF Water, Sanitation and Hygiene, UNICEF WASH in Schools, USAID Environmental Health Project, WASHplus, and World Bank Water and Sanitation Program), and hand searching other relevant reviews. The search string was: ((feces OR faeces) AND sanitation) AND (use or coverage or community or utilization or indicators or household or household characteristics), and included published, unpublished, in press, and grey literature in English, Spanish, Portuguese, French, German, or Italian. 11 studies assessed an intervention's effect on household-level latrine use. 24 household-based and school-based studies, mostly observational or qualitative, assessed sanitation facility characteristics and found increased latrine use was usually associated with latrine functionality, accessibility, cleanliness, privacy, being newer or well maintained, and having amenities that enable hygiene behaviours. None of the sanitation interventions reviewed specifically aimed to increase latrine use among households that already owned latrines. Little is known about how to increase use among non-users in latrine-owning households. A Cochrane review assessed the effect of safe disposal of child faeces on diarrhoea and soil-transmitted health infections, identifying a need for rigorous evaluations of different hardware (eg, potties) and software interventions (eg, education) to improve safe disposal of child faeces. Concurrent with the present study, three other studies assessed the effect of theory-driven interventions on latrine use in rural India. Two reported modest increases in latrine use and one also aimed to improve safe child faeces disposal but no effect was found.

**Added value of this study**

Following a multilevel behaviour change intervention aimed at increasing latrine use and safe disposal of child faeces among latrine-owning households, reported latrine use among individuals in latrine-owning households increased only modestly. Safe disposal of child faeces, which includes both child latrine use and safe disposal by caregivers, increased markedly. We found substantial increases for safe disposal of child faeces by caregivers for both male and female children, and for male child latrine use. Our results show that a low-cost, low-intensity, theory-driven intervention was particularly effective in changing caregiver disposal behaviour, justifying further investment in improving safe disposal of child faeces, especially in rural India where rates remain low across the country. Further, the intervention changed child sanitation behaviour, which has not been a focus of sanitation programming to date.

**Implications of all the available evidence**

Together with existing evidence, our study showed that a multi-level, theory-driven intervention only modestly increased individual latrine use among latrine-owning households in rural India. Importantly, the intervention resulted in increases in safe disposal of child faeces, driven largely by increases in safe disposal of child faeces by caregivers. This intervention was delivered in the final years of the Government of India's Swachh Bharat Mission (SBM) campaign, which invested heavily in increasing latrine coverage to make India open-defecation free. Evidence of theory-driven, behavioural interventions on latrine uptake is mixed. In this study, increases observed were modest, although achieved in the final years of SBM, which probably drove increases observed in control groups. The potential for theory-driven interventions to increase latrine use cannot be ruled out, and environmental barriers must be simultaneously addressed. Further research is needed to understand the effect of theory-driven interventions designed to increase latrine use in other contexts. Safe disposal of child faeces was not integrated into SBM or considered when determining the country's open-defecation free status. Our study showed that improving safe disposal of child faeces among latrine-owning households is both needed and possible. Further investment is warranted, in India and in other contexts where rates are low, to adapt and scale interventions to prevent environmental faecal contamination and subsequent risks to health due to unsafe disposal of child faeces.

We assessed a low-cost, multi-level, theory-driven intervention designed to increase individual latrine use and safe disposal of child faeces among latrine-owning households in rural Odisha, India.

## Methods

### Study design and participants

This cluster-randomised controlled trial was done in 66 rural villages (clusters), in Puri district, Odisha, India (appendix p 2). Details of the trial design, intervention, setting, and complementary qualitative activities are reported elsewhere.^13^ Activities were carried out across three blocks accessible to the research team and intervention delivery team, Rural Welfare Institute (RWI). Among households in rural Puri in 2015–16, 37% had an improved sanitation facility, 94% had an improved drinking water source, and 94% had electricity.^14^

To be eligible for inclusion, villages could not have been declared open-defecation free by the Government or be adjacent to another included village (to prevent spillover effects). To adequately power our study and enable intervention delivery at low cost (average of US$20 per household or less, as specified by the funder), we targeted villages with a mean latrine coverage rate of 60% and a mean size of 100 households (targeting between 50 and 150 households). We also sought villages that were not geographically dispersed (eg, had many or spread-out hamlets or subsections). From June 1 to July 24, 2017, we did a rapid assessment of 282 villages to create an initial sampling frame, with estimates of village size and latrine coverage. From Oct 30 to Dec 30, 2017, we mapped 115 villages presumed to be eligible, and an additional 15 that were not initially assessed, to confirm size, coverage, and eligibility. We identified 66 villages as eligible, including some outside of ideal target coverage and size ranges because we could not identify enough within those ranges that also met eligibility criteria. We analysed mapping data to confirm that the study was adequately powered for planned analyses.

From Jan 30 to April 25, 2018, we censused all households across the 66 villages. All latrine-owning households, regardless of latrine functionality, were eligible to participate in the baseline survey. From Nov 20, 2018, to Feb 18, 2019, all villages were re-censused to confirm latrine ownership status of all households, and all latrine-owning households were eligible to participate in the final survey.

**Figure 1:**
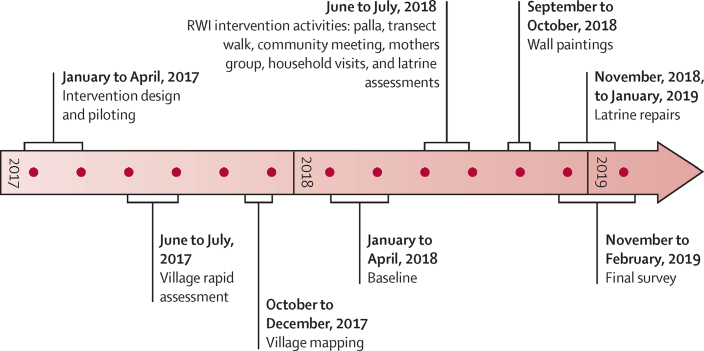
Intervention delivery and data collection timeline

All interactive intervention activities concluded on July 13, 2018, 4 months before the start of endline data collection in November. The community wall paintings finished 1 month before endline (Oct 23, 2018), delayed because of the rainy season. Of the households selected to receive latrine repairs, repairs were delayed because of time needed for assessments and were completed between November, 2018, and January, 2019. Repairs finished 1–7 weeks before endline administration (mean 3 weeks; figure 1).

The Emory University Institutional Review Board (Atlanta, GA, USA; 00098293), the London School of Hygiene & Tropical Medicine (London, UK; 14415), and the Xavier Institute of Management (Bhubaneswar, Odisha, India; 131216) ethics review committees approved study protocols. Participants provided oral consent before data collection. The protocol has been published.^13^

### Randomisation and masking

We grouped eligible villages into four strata on the basis of village size and latrine coverage. A research team member (CLN), who was not involved in data collection or intervention delivery, conducted stratified block randomisation to assign villages to control or intervention groups using the randomize package in Stata (version 15).

Enumerators were not told village assignments before baseline. Neither participants nor field assessors were masked to study group assignment because it was not possible. Participants were not told that the study aimed to assess the intervention. The data collection team was different from the intervention delivery team.

### Procedures

#### Intervention design and delivery

The research team designed the intervention between January and April, 2017. We conducted formative research to identify factors influencing latrine use and safe disposal of child faeces, including the generation of problem and solution trees with our local partner Bhabagrahi Kala Niketan and community stakeholders. We identified the following barriers: (1) non-functional latrines, (2) lack of practical knowledge regarding latrine use and safe disposal, (3) preference for open defecation, (4) lack of understanding of latrine use and disposal benefits, (5) unsuitable design, and (6) inaccessible water. We categorised barriers into the types of behavioural factors they represented, leveraging an expanded version of the behaviour-centred design checklist^15^ that also included factors from the COM-B^16^ and RANAS^17^ models of behaviour change. This categorisation allowed identification of the appropriate behaviour change techniques to integrate into the intervention activities that would target the desired behavioural factors.^18^

The multi-level intervention, which reiterated the motto *moro swacha, sustha, sundara grama* (our clean, healthy, beautiful community), included activities at the community, subgroup (caregivers of children aged 5 years and younger), and household levels. The intervention aimed to influence latrine use by addressing barriers 1–4 and safe child faeces disposal by addressing barriers 2 and 4. We could not address water inaccessibility or latrine design while also balancing the policy-relevant requirement to spend less than $20 on average per household. Table 1 describes each intervention activity, of which activities 2–9 were pilot tested to improve the intervention on the basis of community member feedback. Additional information about intervention design is available elsewhere.^19^

**Table 1:** Intervention activities, target behavioural factors, participants, and implementers

	**Description and aim**	**Behavioural factors targeted**	**Target participants**	**Implementer**
Preliminary community visit	Preintervention visit with community leaders to build rapport, plan activity logistics (dates, timing, and locations), and learn about relevant community dynamics	NA	Community leaders	RWI community mobilisers
Palla	Comedic folk theatre performance with a series of sanitation-based skits to engage community members on the health and many non-health benefits of latrine use; risks and costs associated with open defecation; and provide action knowledge on latrine use, pit emptying, and safe disposal of child faeces	Motivation (comfort, status, justice, and nurture); injunctive norms; perceived risk and vulnerability; action knowledge	All community members	Two palla troupes
Coloured powder transect walk	Early morning walk with community members to encourage a re-evaluation of village cleanliness by marking faeces with coloured powder throughout the village and known defecation fields. Walks ended with a handwashing demonstration and group discussion	Motivation (disgust); remembering	All community members	RWI community mobilisers
Community meeting	Interactive meeting with facilitated group discussion on sanitation problems and solutions, creation of and commitment to a community action plan for sanitation goal, and celebration of households whose members already exclusively use a latrine for defecation (positive deviants)	Barrier planning; action planning; commitment; motivation (comfort, status, and justice)	All community members	RWI community mobilisers
Mothers' meeting	Interactive meeting with discussion on the health risks of unsafe disposal of child faeces, demonstration and guided practice on safe management of child faeces and the use of hardware to enable safe faeces disposal (each caregiver was provided a plastic scoop and potty), and group pledge towards practicing safe disposal	Perceived risk and vulnerability; action knowledge; physical opportunity; motivation (nurture); descriptive norms	Primary caregivers of children aged 5 years and younger (regardless of latrine ownership*)	RWI community mobilisers
Recognition banners	Banner hung outside home of households whose members already exclusively use a latrine for defecation (positive deviants) identified during community meeting to publicly acknowledge and celebrate the household	Descriptive norms; injunctive norms; motivation (status); remembering	All community members	RWI community mobilisers
Household visit	Visit with household members to reiterate and reflect upon key intervention messages and facilitate household commitment towards exclusive latrine use, with distribution of a reminder poster	Commitment; remembering	Latrine-owning households	RWI community mobilisers
Community wall painting	Public wall painting that displays the community meeting action steps and a map of the village that identifies which households have a latrine and which already exclusively use a latrine for defecation (positive deviants), to track progress towards the community sanitation goal	Injunctive norms; descriptive norms; remembering; motivation (status)	All community members	Two local artisan groups
Latrine assessment and repairs	Assessment of latrine condition and subsequent provision of basic repairs, as needed, to ensure functionality and privacy—eg, fixing I-pipe connection to pit, repairing door frame, and cementing slab cover to top ring to prevent pit from filling with rainwater	Physical opportunity	Latrine-owning households in need of repairs	Assessors from Emory University and two local contractors

Intervention activities are listed in the order they were implemented. NA=not applicable. RWI=Rural Welfare Institute.

* Primary caregivers who did not have a latrine in their household were provided information on how to safely bury their child's faeces.

RWI hired and managed community mobilisers, who worked in four teams of five (one supervisor, four mobilisers), to lead transect walks, community meetings, mothers’ meetings, and household visits. Each team carried out activities in eight to ten villages. Each mobiliser was provided with a manual with step-by-step instructions for each activity. PR and GDS conducted 12-day training, including 5 days reviewing activities and 7 days of field practice, and facilitated debriefs after each practice to provide feedback and determine final changes to activities.

Two local troupes carried out the pallas. They were provided with scripts for the six sanitation-based skits, and practiced for RWI and Emory staff and in non-trial villages. Two local artisan groups completed the community wall paintings that were based on hand-drawn maps made by the community mobilisers with assistance from village leaders. Two contracting groups repaired latrines after conducting assessments of latrines identified from baseline data as potentially in need of repairs. Repairs focused on functionality and privacy.

### Data collection

A Puri-based, Odia-speaking team of 13 enumerators (12 female and one male) and two female supervisors carried out data collection; PR managed all field activities. Data were collected using ODK Collect. Before baseline and endline, enumerators attended week-long trainings, including 3 days practicing in pilot villages. Survey tools were developed in English then translated into Odia (by PR). Team members provided feedback on translation and content during training and piloting.

Each household in enrolled villages was assigned a unique identification number, which was noted on a hand-drawn map. Maps were used again at endline to confirm identification and enable linkage to baseline data. At each household, enumerators assessed eligibility, consented those eligible and willing, and administered the survey. Participants provided information about household demographics, latrine use, faeces disposal behaviours, and water and sanitation facilities. Female household heads were the primary survey respondent given their presumed ability to accurately report household members’ defecation behaviours.^11^ Enumerators then did spot check observations of household latrines to note conditions. At endline, participants were asked about intervention activities at the end of the survey (after latrine-use questions). If no household members were present, enumerators recorded latrine ownership if visual confirmation was possible.

### Outcomes

We specified two primary outcomes: (1) latrine use by each household member aged 5 years and older, and (2) safe disposal of child faeces for children younger than 5 years. At baseline, participants listed all household members (at endline, this list was automatically generated using the baseline data and was verified by the respondent). For each household member the enumerator asked: “The last time [NAME] defecated, did [NAME] defecate in the open or use the latrine?”. Response options included: “open”, “latrine”, or “somewhere else (eg, potty, nappy, bedpan)”. If present, household members aged 18 years or older were asked to answer for themselves. For household members younger than 5 years, if the defecation place was not the latrine, the following was asked: “What was done to dispose of the stool?”. We also report latrine spot checks as observed by field staff to identify whether the latrine appeared to be in use (eg, water on platform, pan colour, smell, water container present, faecal remnants, etc).

For the primary outcome of safe disposal of child faeces, we report as per the WHO/UNICEF Joint Monitoring Programme for Water Supply, Sanitation, and Hygiene (JMP) definition,^20^ which includes both child latrine use and caregiver disposal of child faeces directly into the latrine. We also separately report child latrine use and caregiver disposal of child faeces into the latrine because these are two distinct behaviours with different actors.

As a secondary outcome, we assessed latrine ownership among all village households at baseline and endline to assess change in latrine coverage during the follow-up period.

### Statistical analysis

The trial sample size of 66 villages was based on reported latrine use at last defecation. We used a Monte Carlo simulation approach to estimate sample size, which accounted for the inclusion of baseline latrine use in our models, adjustment for within-person and within-cluster correlation, and unbalanced cluster sizes.^21^ Based on sanitation trial data collected from 2011 to 2013 in the same rural district of Odisha,^22^ we assumed a baseline latrine use of 45%, a village-level intracluster correlation coefficient (ICC) of 0·10, and a within-person correlation from baseline to follow-up of 0·60. No previous trials had assessed the effect of an intervention on latrine use. We concluded that a 10% increase in absolute use was reasonable, given that post-hoc analyses found that every 10·0% increase in coverage resulted in a 5·8% increase in use.^6^ Assuming a mean of 292 eligible participants per village (cluster size coefficient of variation 0·35), 10% loss to follow-up, 80% power, and significance level of 0·05, we determined that a sample size of 33 villages per group was required. No allowance was made in sample size calculations for co-primary endpoints.

We did an intention to treat analysis, using the difference-in-differences (DID) approach (as noted in our pre-analysis plan [RIDIE-STUDY-ID-5a12600105509]) to estimate the intervention effect adjusted for baseline differences in latrine use and safe disposal of child faeces and temporal trends unrelated to the intervention activities. The DID model of latrine use was fitted using the following model specification, where the coefficient β_3_(time*treatment) is the estimate of the causal effect:

$$\text{latrine}\text{use}=\beta_{0}+\beta_{1}({\text{treatment}}_{\text{assignment}})+\beta_{2}(\text{time})+\beta_{3}(\text{time}*\text{treatment})+\beta_{4}(\text{age})+\beta_{5}(\text{sex})+\beta_{6}(\text{male educational attainment})+\beta_{7}(\text{female educational attainment})+\beta_{8}(\text{household size})+\beta_{9}(\text{household SES})$$

The DID model of latrine use was adjusted for participant age (5–12 years, 13–19 years, 20–29 years, 30–39 years, 40–49 years, 50–59 years, and ≥60 years), sex, years of education of male head of household, years of education of female head of household, number of people residing in household, and household socioeconomic status (SES). To measure household SES, we constructed an asset index by combining information on asset ownership (watch or clock, pressure cooker, telephone, refrigerator, chair, mattress, cot, table, electric fan, water pump, motorbike, thresher, tractor, television, and electricity) using polychoric principal component analysis.^23^ Study households were divided into five SES quintiles on the basis of asset index score (quintile 1 was the lowest SES, quintile 5 the highest). We compared the results of covariate adjusted models with those of unadjusted models as a sensitivity analysis.

DID models were estimated using generalised estimating equations with an exchangeable correlation structure and robust SEs to account for the clustering of observations within villages.^24^, ^25^ To avoid the issues related to estimation and interpretation of the treatment effect in non-linear DID models,^26^ we fitted heteroskedasticity-adjusted linear probability models, which allow for direct interpretation and testing of the interaction coefficient as the DID term.^27^ We ensured that the marginal probabilities of the outcome for each time*treatment combination fell within the unit interval [0,1], and did sensitivity analyses by fitting equivalent logit models. We found no substantive difference in the magnitude or significance of the treatment effect estimated in linear probability models and those derived from the cross-difference of marginal probabilities in logit models. We report the model coefficients from the linear probability models for ease of interpretation.

For the outcomes of safe disposal of child faeces we fitted similarly specified models, excluding the covariate for age. We did not make multiplicity adjustments for these measures given the small number of outcomes and the lack of statistical consensus regarding the appropriate use of these adjustments.^28^ We fitted sex-stratified models of all outcomes to examine sex-related differences in the effect of the intervention.

To examine the robustness of our findings to potential self-report bias, we fitted a household-level DID model to examine if a difference existed between study groups in enumerator perception of whether latrines looked to be in use, recorded during the spot-check observation.

Repeated surveys have been posited to influence overreporting, justifying study designs that include different households at endline than at baseline.^29^ In our sample, 578 households (278 [48%] in the intervention group and 300 [52%] in the control group) were re-surveyed shortly after baseline to assess differences in open defecation reporting when using different questions.^30^ To assess whether repeated surveys affected outcomes, we reanalysed our primary outcome models with a covariate indicating whether or not the household participated in this additional nested measurement survey.

We did not use imputation methods to adjust for missingness. We did several tests and sensitivity analyses to examine the potential effect of missing data on reported effect estimates (appendix p 3). Data were analysed using Stata (version 16). This trial is registered with ClinicalTrials.gov, NCT03274245 and the analysis plan is registered with RIDIE (5a12600105509).

### Role of the funding source

International Initiative for Impact Evaluation (3ie) reviewed and approved the study protocol but had no role in study design, data collection, data analysis, data interpretation, or writing of the report. 3ie did influence agreement on common outcome measures to enable comparison across their funded investigations.

## Results

**Figure 2:**
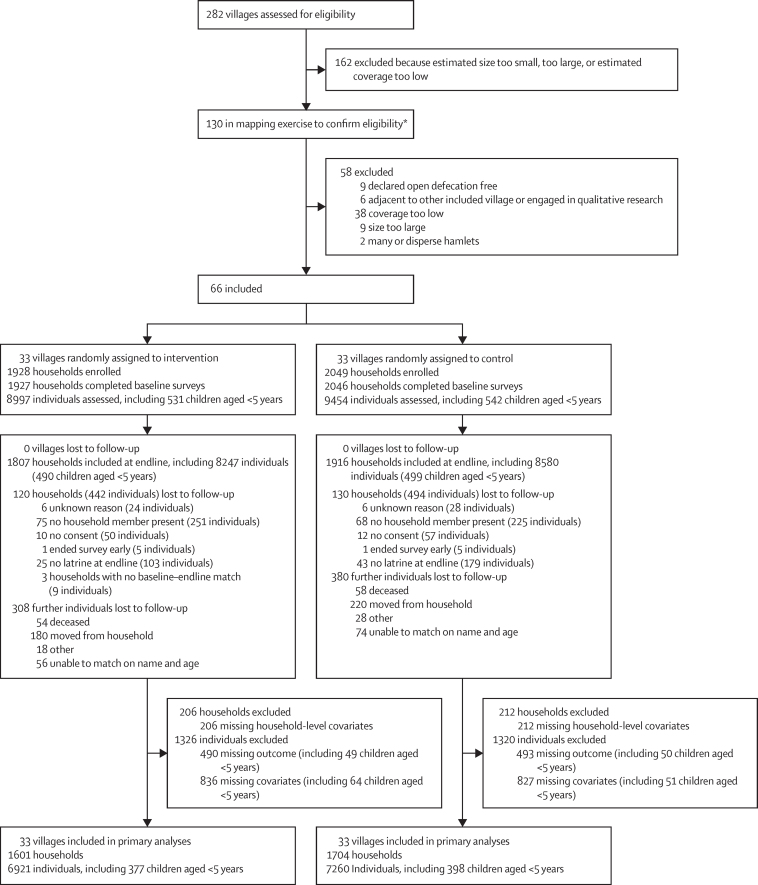
Trial profile *Included 15 villages not included in initial assessment.

We randomly assigned 66 villages (33 [50%] villages to the intervention group, 33 [50%] as controls; figure 2). In intervention villages, we censused 2846 households, representing 12 950 individuals. 1928 (67·7%) households with at least one latrine were eligible to participate (mean latrine coverage at village level 68·2% [SD 14·5]). At baseline, 8997 individuals from 1927 latrine-owning households were enrolled. In control villages, we censused 3017 households, representing 13 400 individuals; 2049 (67·9%) households with at least one latrine were eligible to participate (mean latrine coverage at village level 67·5% [SD 11·0]). At baseline, 9454 individuals from 2046 latrine-owning households were enrolled.

Baseline and endline data were available for 14 181 individuals (6921 [48·8%] in the intervention group and 7260 [51·2%] in the control group), including 13 406 (94·5%; 6544 [48·8%] in the intervention group and 6862 [51·2%] in the control group) aged 5 years and older to assess change in latrine use, 774 (5·5%; 377 [48·7%] in the intervention group and 397 [51·3%] in the control group) to assess JMP-defined safe disposal, 772 (5·4%; 376 [48·7%] in the intervention group and 396 [51·3%] in the control group) to assess latrine use in children younger than 5 years, and 406 (2·9%; 199 [49·0%] in the intervention group and 207 [51·0%] in the control group) to assess safe disposal of child faeces by caregivers. Baseline demographic and water and sanitation characteristics were balanced across groups (table 2). Among the analytic sample, the village-level ICC for latrine use was 0·094 at baseline.

**Table 2:** Census and eligible baseline population study characteristics

		**Intervention**	**Control**	**Total**
Villages		33	33	66
Households censused		2846	3017	5863
Individuals represented in census		12 950	13 400	26 350
Households per village		86·2 (25·4)	91·4 (24·6)	88·8 (25·0)
Population censused per village		392·4 (115·3)	406·1 (119·6)	399·2 (116·7)
Eligible households (ie, own a latrine)*		1928	2050	3978
Households enrolled		1927	2046	3973
Individuals in enrolled households		8997	9454	18 451
	Female respondent	1665 (86·4%)	1764 (86·2%)	3429
	Male respondent	262 (13·6%)	282 (13·8%)	544
Religion of household				
	Hindu	1853 (96·2%)	1941 (94·9%)	3794
	Muslim	42 (2·2%)	72 (3·5%)	114
	Other†	26 (1·3%)	24 (1·2%)	50
	No religion	6 (0·3%)	9 (0·4%)	15
Caste or tribe of household‡				
	General	705 (36·6%)	663 (32·4%)	1368
	Scheduled caste	280 (14·5%)	228 (11·1%)	508
	Other backward caste	694 (36·0%)	899 (44·0%)	1593
	Scheduled tribe	25 (1·3%)	10 (0·5%)	35
	Other or unknown	223 (11·6%)	246 (12·0%)	469
Government subsidies				
	BPL	143 (7·4%)	181 (8·8%)	324
	Antyodaya	121 (6·3%)	108 (5·3%)	229
	Ration card	753 (39·1%)	855 (41·8%)	1608
	Combination BPL, antyodaya, or ration card§	418 (21·7%)	404 (19·7%)	822
	None or unknown	492 (25·6%)	498 (24·3%)	990
Education of male head of household				
	Anganwadi	76 (3·9%)	66 (3·2%)	142
	Primary	426 (22·1%)	445 (21·8%)	871
	Upper primary	343 (17·8%)	360 (17·6%)	703
	Secondary	527 (27·3%)	570 (27·8%)	1097
	Senior secondary	77 (4·0%)	84 (4·1%)	161
	Graduate or postgraduate	112 (5·8%)	115 (5·6%)	227
	Never attended	184 (9·5%)	218 (10·7%)	402
	Unknown	108 (5·6%)	105 (5·1%)	213
	No male head	74 (3·8%)	83 (4·1%)	157
Education of female head of household				
	Anganwadi	60 (3·1%)	65 (3·2%)	125
	Primary	559 (29·0%)	560 (27·4%)	1119
	Upper primary	306 (15·9%)	330 (16·1%)	636
	Secondary	301 (15·6%)	350 (17·1%)	651
	Senior secondary	40 (2·1%)	51 (2·5%)	91
	Graduate or postgraduate	40 (2·1%)	25 (1·2%)	65
	Never attended	561 (29·1%)	604 (29·5%)	1165
	Unknown	35 (1·8%)	34 (1·7%)	69
	No female head	25 (1·3%)	27 (1·3%)	52

Data are n, n (%), or mean (SD). BPL=below poverty line.

* True total eligible might be larger. 526 households did not participate in census because not home, not available, or ended census before latrine assessment.

† Includes Hindu and Muslim (n=2 in the intervention group), Hindu and other (n=2 in the control group), Christian (n=1 in the intervention group), Buddhist and Neo Buddhist (n=1 in the intervention group and n=1 in the control group), and other unnamed religion (n=22 in the intervention group and n=21 in the control group).

‡ We present the term other backward castes as defined by the Government of India to classify a section of population that are educationally or socially disadvantaged. The term is one of the official classifications along with general, scheduled castes, and scheduled tribes.

§ Combination may include BPL and antodaya; BPL, antodaya, and ration card; BPL and ration card; or antodaya and ration card.

At endline, 1405 (87·8%) respondents in intervention households reported that latrine-use promotion activities occurred in their village in the previous 6 months, compared with only 50 (2·9%) in control villages. Among latrine-owning households in the intervention group, interaction with intervention activities was mixed. 1250 (69·2%) respondents had awareness of the intervention motto, 1267 (70·1%) reported attendance at the palla performance, and 1476 (81·7%) participated in household visits. 777 (43·0%) respondents attended the community meeting, 532 (29·4%) attended the transect walk. and only 620 (34·3%) reported seeing the wall painting. 309 (59·8%) households with children younger than 5 years attended the mothers’ meeting (table 3). Of those selected for repairs (n=382) and surveyed at endline (n=358), 268 (74·8%) reported receiving repairs. The most common repairs were door (155 [57·0%]), flooring (57 [21·0%]), pit lining (52 [19·1%]), slab cover (40 [14·7%]), and pipe connection (38 [14·0%]; appendix p 3).

**Table 3:** Self-reported engagement in intervention activities

		**All intervention households surveyed at endline, regardless of latrine status (n=2828)**	**Intervention households surveyed at baseline and endline with a latrine at both rounds (n=1807)**
Respondent recalls hearing intervention motto		1859 (65·7%)	1250 (69·2%)
Someone in household attended			
	Palla	1851 (65·5%)	1267 (70·1%)
	Transect walk	775 (27·4%)	532 (29·4%)
	Community meeting	1088 (38·5%)	777 (43·0%)
	Mothers' meeting (among all households)	727 (25·7%)	503 (27·8%)
	Mothers' meeting (among households with children aged <5 years)*	441 (58·0%)	309 (59·8%)
Household visit conducted			
	Yes	1813 (64·1%)	1476 (81·7%)
	Refused visit	21 (0·7%)	6 (0·3%)
Respondent has seen wall painting		949 (33·6%)	620 (34·3%)

Data are n (%).

* 760 households with children younger than 5 years in all households were surveyed at endline; 517 households with children younger than 5 years were surveyed at baseline and endline and had a latrine at both rounds. Households with children younger than 5 years at the time of the intervention but aged 5 years at endline were not captured in this number.

A greater proportion of females than males in both groups reported using latrines at endline (appendix p 4). In intervention villages, the greatest increase in use was among 50–59-year-olds (23·1 percentage points) and the smallest was among 20–29-year-olds (17·3 percentage points) and 30–39-year-olds (17·2 percentage points; appendix p 5).

In fully adjusted DID models, reported latrine use for individuals aged 5 years and older increased in the intervention group at endline by 6·4 percentage points (95% CI 2·0–10·7) among all individuals (6·6 percentage points [2·2-11·0] among females; 6·1 percentage points [1·4–10·8] among males; table 4; appendix pp 6–8). We found an increase of 4·3 percentage points (1·0–8·0) in the proportion of latrines appearing to be in use, as observed by enumerators, among the intervention households at endline (appendix p 9). We found no statistically significant association between reported latrine use and household participation in the nested measurement survey (p=0·70; appendix p 10), indicating no effect of this nested survey on the primary outcome. Unadjusted models did not substantively differ from fully adjusted models (appendix p 11).

**Table 4:** Effect of intervention by outcome

		**N**		Baseline*		Endline*		**Percentage point difference (95% CI)**†
		Intervention	Control	Intervention	Control	Intervention	Control	
Latrine use								
	All individuals aged ≥5 years	6544	6862	3954 (60·4%)	4231 (61·7%)	5267 (80·5%)	5170 (75·3%)	+6·4 (2·0 to 10·7)
	Males aged ≥5 years	3281	3385	1837 (56·0%)	1884 (55·6%)	2511 (76·5%)	2373 (70·1%)	+6·1 (1·4 to 10·8)
	Females aged ≥5 years	3263	3477	2117 (64·9%)	2347 (67·5%)	2756 (84·5%)	2797 (80·4%)	+6·6 (2·2 to 11·1)
Latrines appearing to be in use‡		1591	1690	1150 (72·3%)	1234 (73·0%)	1340 (84·2%)	1363 (80·7%)	+4·3 (0·6 to 8·0)
JMP-defined safe disposal of child faeces§								
	All children aged <5 years	377	397	87 (23·1%)	86 (21·7%)	226 (60·0%)	172 (43·3%)	+15·2 (7·9 to 22·5)
	Males aged <5 years	182	189	39 (21·4%)	37 (19·6%)	110 (60·4%)	72 (38·1%)	+20·4 (11·3 to 29·7)
	Females aged <5 years	195	208	48 (24·6%)	49 (23·6%)	116 (59·5%)	100 (48·1%)	+10·4 (0·2 to 20·5)
Latrine use among children aged <5 years								
	All children aged <5 years	376	396	58 (15·4%)	72 (18·2%)	157 (41·8%)	148 (37·4%)	+7·1 (0·4 to 13·9)
	Males aged <5 years	182	188	23 (12·6%)	27 (14·4%)	80 (44·0%)	61 (32·4%)	+13·2 (3·8 to 22·7)
	Females aged <5 years	194	208	35 (18·0%)	45 (21·6%)	77 (39·7%)	87 (41·8%)	+1·5 (−8·0 to 10·9)
Safe disposal of child faeces by caregivers								
All children aged <5 years		199	207	12 (6·0%)	7 (3·4%)	67 (33·7%)	22 (10·6%)	+20·4 (11·6 to 29·2)
	Males aged <5 years	95	103	7 (7·4%)	5 (4·9%)	29 (30·5%)	9 (8·7%)	+19·3 (8·5 to 30·0)
	Females aged <5 years	104	104	5 (4·8%)	2 (1·9%)	38 (36·5%)	13 (12·5%)	+21·2 (9·1 to 33·2)
Latrine ownership at endline among non-owners at baseline		848	887	NA	NA	187 (22·1%)	193 (21·8%)	+0·3 (−8·76 to 9·35)

Data are n (%), unless otherwise indicated. JMP=WHO/UNICEF Joint Monitoring Programme for Water Supply, Sanitation, and Hygiene.

* Baseline and endline proportions are unadjusted.

† Intention-to-treat analysis. Effect sizes were derived from fully adjusted models, including adjustment for clustering.

‡ Assessed from enumerator perception of latrine being in use during spot-check observation.

§ Safe disposal of child faeces, as per JMP definition, includes both latrine use by children younger than 5 years and safe disposal of child faeces by caregivers. For this analysis, we included all children younger than 5 years at baseline.

In fully adjusted DID models, JMP-defined safe disposal of child faeces, which considers both child latrine use and caregiver disposal, increased in the intervention group at endline by 15·2 percentage points (95% CI 7·9–22·5). Sex-stratified analysis showed that JMP-defined safe disposal increased in the intervention group at endline more for boys (20·4 percentage points [11·3–29·7]) than for girls (10·4 percentage points [0·2–20·5]; table 4; appendix pp 12–14). Disaggregating, we found that both child latrine use and caregiver safe child faeces disposal increased in the intervention group at endline, but differently (appendix pp 14–15, 17).

In fully adjusted DID models, child latrine use increased in the intervention group at endline by 7·1 percentage points (95% CI 0·4 to 13·9). Sex-stratified analysis showed that child latrine use increased in the intervention group at endline much more for boys (13·2 percentage points [3·8 to 22·7]) than for girls (1·5 percentage points [–8·0 to 10·9; table 4; appendix pp 14–16). In the intervention group, proportional increases in latrine use were greater among boys than among girls (31·4 percentage points *vs* 21·7 percentage points) and older children (40·2 percentage points among those aged 24–35 months, 31·6 percentage points among those aged 36-47 months, and 39·5 percentage points among those aged 48–59 months; appendix p 5).

In fully adjusted DID models, safe disposal of child faeces by caregivers increased in the intervention group at endline by 20·4 percentage points (95% CI 11·6–29·2). Sex-stratified results were similar for boys (19·3 percentage points [8·5–30·0]) and girls (21·2 percentage points [9·1–33·2]; table 4; appendix pp 14, 17–18). In the intervention group, proportional increases for safe disposal of child faeces by caregivers were greater among girls than among boys (31·7 percentage points *vs* 23·1 percentage points) and the youngest children (27·7 percentage points among those aged 0–12 months and 31·4 percentage points among those aged 13–23 months; appendix p 5).

Unadjusted models did not substantively differ from fully adjusted models for JMP-defined safe disposal of child faeces or safe disposal of child feces by caregivers (appendix p 11). The effect of the intervention on latrine use among children younger than 5 years was statistically significant in the adjusted model (p=0·039) but not in the unadjusted model (p=0·057; appendix p 11).

We found no difference between intervention and control groups in the proportion of households without latrines at baseline who reported having one at endline (table 4). Endline latrine coverage was similar between groups (table 4).

We found a similar pattern of missingness in both study groups and within covariate strata, and the results of sensitivity analyses showed no evidence of bias in effect estimates resulting from the exclusion of participants with missing data. During the trial, no adverse events were reported.

## Discussion

The multi-level behaviour change intervention increased JMP-defined safe disposal of child faeces and safe disposal of child faeces by caregivers, but had only modest effects on reported latrine use. Three other studies assessing the effect of behaviour change interventions on latrine use in rural India occurred concurrently. Similar increases in reported latrine use among individuals aged 5 years and older were found in Karnataka (4·6%)^31^ and Gujarat (4·6%),^29^ while the study in Bihar^32^ reported null effects. Only the intervention in Karnataka aimed to increase safe disposal of child faeces, and found no effect.^33^ Collectively, these studies show the challenge of achieving the changes in sanitation practices that are necessary to prevent faecal exposures and improve health.

The modest effect on latrine use might be because the behavioural factors that were targeted by the intervention activities were insufficient to change behaviours of those still not using latrines. Time and cost constraints prevented the intervention from addressing all known behavioural factors, notably water access and latrine design. Difficulty accessing water and discontent regarding latrine design are known barriers to latrine uptake in Odisha.^9^, ^11^ Individuals with government-funded latrines are more likely to open defecate than those with privately constructed latrines, plausibly because of design features, such as the smaller pit sizes.^34^ Participants who engaged in qualitative research in non-trial villages that received the intervention agreed, expressing that latrine uptake was not possible if water and design issues were not addressed.^35^ These reflections about the physical environment are consistent with the behaviour change work by Aunger and Curtis,^16^ who describe the need for appropriate behavioural settings, and Michie and colleagues,^17^ who write that appropriate physical opportunities, or environmental conditions, are needed to make possible or prompt behaviours.^16^ Our intervention did address the physical environment by repairing non-functional (eg, broken pipes) and non-private (eg, missing doors) latrines, but improving water access and design was not possible. Government-provided latrines might have furnished a sufficient environment for some, but not all. In the absence of accommodating facilities, even the most perfect behaviour change interventions cannot expect high uptake, which is especially true among those who are resistant to change and tend to be committed to their traditions and habits.^36^

Reported latrine use increased in both study groups, but how increases were realised and if they can be sustained remains unclear. Only 2·9% of respondents from control villages reported any kind of latrine promotion activities occurring in their village since baseline. If SBM is responsible for the increases**,** village activities are unlikely to be the cause. Television, radio, internet, newspapers, or other media could be an explanation, and so could coercion. SBM was reported to use coercion, including harassment, fines, detention, and denial of benefits, to encourage latrine construction and use.^7^ Coercion occurred in the study area. Qualitative research found that a woman caught open defecating was threatened with losing her ration card.^35^ The winter season might also have influenced endline latrine use across groups.^9^ Regardless of how increases were achieved, understanding if they are sustained and if the strategies used by the Government are harmful is essential.

The increases in JMP-defined safe disposal of child faeces seem driven by the substantial increase in safe disposal of child faeces by caregivers. Our findings highlight that caregiver disposal is a distinct behaviour within JMP-defined safe disposal of child faeces that requires and is responsive to tailored behaviour change strategies. The strategies used in our intervention were effective and should be adapted and scaled-up in India and other contexts, particularly where rates of safe disposal of child faeces by caregivers are low.

This study had several limitations, particularly the constraints placed on the intervention cost and the period for follow-up. Although the $20-per-household limit was deemed to be necessary to achieve scale, it severely restricted the nature of the intervention. Moreover, the 4-month period between the completion of the intervention delivery (1 month if considering completion of the wall paintings, and even shorter among select latrine repair recipients) and the single follow-up assessment did not allow us to assess if effects might be sustained over even the medium period, or to assess whether the intervention effects might increase with time. We relied on respondents to report behaviours, which is subject to bias. For all outcomes, we intentionally asked about the last defecation event, which has shown moderate agreement with the data collected from passive latrine use monitors.^37^ Latrine observation data support findings, confirming a greater increase in use among intervention households compared with controls. Further, we found no effect of repeated surveys on latrine use. Whether latrine use behaviour will be sustained, and how slippage, or reversion to open defecation, might occur or differ between groups, is unclear, particularly as the COVID-19 pandemic might influence sanitation-related behaviours.^38^ Finally, neither faecal exposure nor health were investigated. Given the extent that open defecation and unsafe disposal persists, meaningful effects on exposure and health are unlikely.

Unsafe disposal of child faeces, particularly caregiver disposal, continued at high rates in study villages, posing risks to the environment and health. Among those reporting unsafe disposal of child faeces in Bangladesh, an increased prevalence of *Escherichia coli* was found on children's hands and in stored water.^39^ To improve health outcomes, transformative WASH approaches, which are those that seek to radically reduce faecal contamination of household environments, are needed.^40^ Safe disposal of child faeces, including both caregiver disposal and child latrine use, is an essential element of transformative WASH. The Government of India should include child faeces disposal when determining open-defecation-free status of villages. Unsafe disposal of child faeces by caregivers is a cause of environmental contamination necessitating further investment in the delivery of effective interventions to manage safe disposal of child faeces, such as our intervention. India cannot be truly open-defecation free if this crucial behaviour remains ignored.

## Data sharing

All data used in the analysis are available under the project name “Low-cost behaviour change interventions to improve latrine use and safe child faeces disposal in rural Odisha, India” at https://osf.io/bewpz/.

## Declaration of interests

We declare no competing interests.
