# Supplementary material for: Effect of a low-cost, behaviour-change intervention on latrine use and safe disposal of child faeces in rural Odisha, India: a cluster-randomised controlled trial
Source: Lancet Planet Health. 2022 Feb 9;6(2):e110–21. doi: 10.1016/S2542-5196(21)00324-7 (PMC8850376; doi:10.1016/S2542-5196(21)00324-7)
Supplement: Supplementary appendix [file mmc1.pdf]

THE LANCET
Planetary Health

Supplementary appendix

This appendix formed part of the original submission and has been peer reviewed. We post it as supplied by the authors.

Supplement to: Caruso BA, Sclar GD, Routray P, et al. Effect of a low-cost, behaviour-change intervention on latrine use and safe disposal of child faeces in rural Odisha, India: a cluster-randomised controlled trial. *Lancet Planet Health* 2022; **6:** e110–21.

SUPPLEMENTAL MATERIALS

Effect of the *Sundara Grama* behavior change intervention on latrine use and safe disposal of child feces in rural Odisha, India: a cluster randomized controlled trial

Bethany A. Caruso, Gloria D. Sclar, Parimita Routray, Corey L. Nagel, Fiona Majorin, Steven Sola, William J. Koehne III, Thomas Clasen

Bethany A. Caruso,* PhD, Hubert Department of Global Health, Rollins School of Public Health, Emory University, 1518 Clifton Rd NE, Atlanta, GA 30322, USA

Gloria D. Sclar, MPH, Gangarosa Department of Environmental Health, Rollins School of Public Health, Emory University, 1518 Clifton Rd NE, Atlanta, GA 30322, USA

Parimita Routray, PhD, Independent Consultant, Bhubaneswar, 751006, India

Corey L. Nagel, PhD, College of Nursing, University of Arkansas for Medical Sciences, Little Rock, Arkansas, USA; College of Public Health, University of Arkansas for Medical Sciences, Little Rock, Arkansas, USA

Fiona Majorin, PhD, Department of Disease Control, Faculty of Infectious and Tropical Diseases, London School of Hygiene and Tropical Medicine, London WC1E 7HT, UK

William J. Koehne III, MPH, Department of Epidemiology, Rollins School of Public Health, Emory University, 1518 Clifton Rd NE, Atlanta, GA 30322, USA

Steven Sola, MSPH, Gangarosa Department of Environmental Health, Rollins School of Public Health, Emory University, 1518 Clifton Rd NE, Atlanta, GA 30322, USA

Professor Thomas Clasen, PhD, Gangarosa Department of Environmental Health, Rollins School of Public Health, Emory University, 1518 Clifton Rd NE, Atlanta, GA 30322, USA

*Correspondence to: Dr. Bethany Caruso, Hubert Department of Global Health, Rollins School of Public Health, Emory University, 1518 Clifton Rd NE, Atlanta, GA 30322, USA.

Telephone: +1 (978) 314 2432 Email: bcaruso@emory.edu

<u>Supplemental</u> Figure 1: Intervention and control villages for evaluation of *Sundara Grama* latrine use and safe child feces disposal intervention, Puri District, Odisha, India

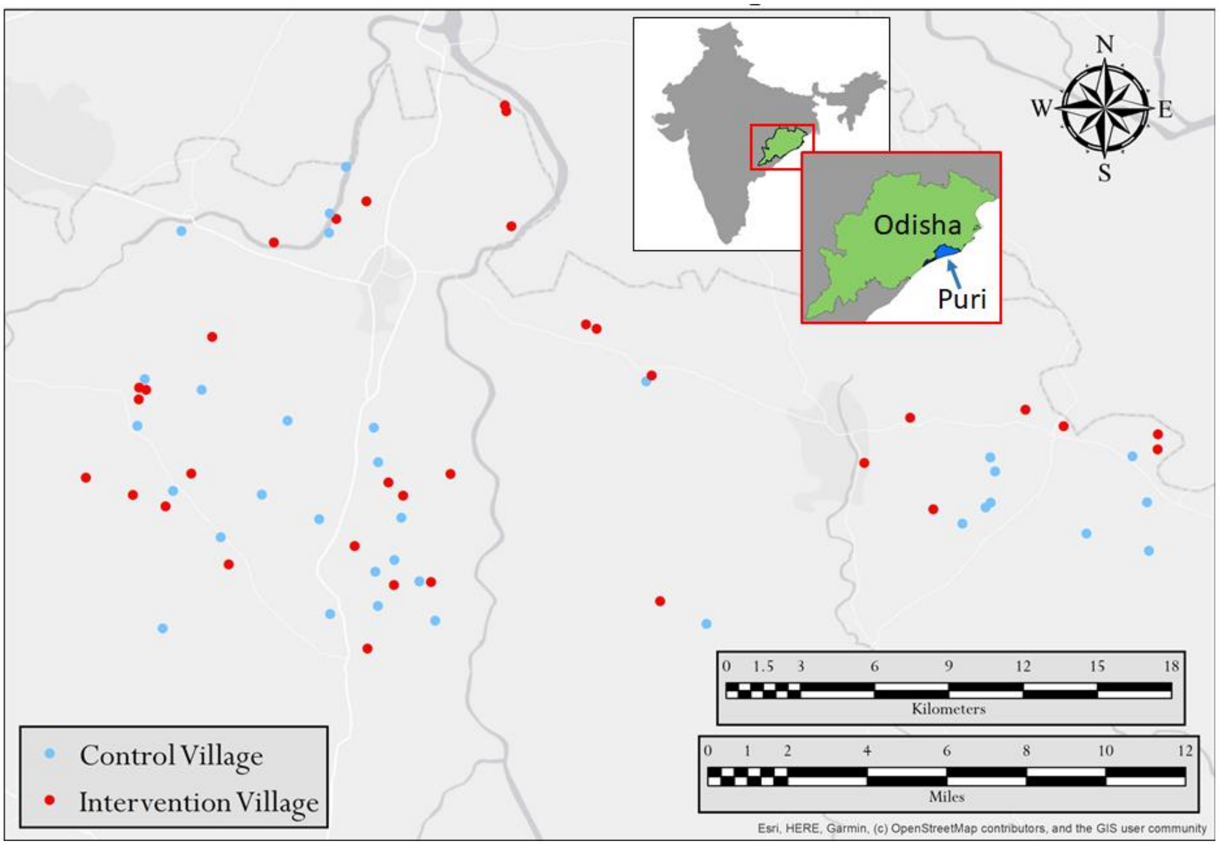

Notes:
The main map was created in ArcMap 10.8.1 by ESRI. It used data from HERE, Garmin, and OpenStreetMap. For the insert, shapefiless for India, Odisha, and Puri were retrievd from the GeoData Library of the University of Texas at Austin: https://geodata.lib.utexas.edu/.

Supplemental Text

‘Missing Data

Cases with missing data were excluded in the reported analyses (i.e. complete case analysis) and we did not use imputation methods to adjust for missingness in our analyses. (The numbers of and reasons for missingness are detailed in the trial flow diagram presented in Figure 2 of the manuscript.)

A number of tests and sensitivity analyses were conducted to examine the potential impact of missing data. These included 1) examination of the distribution of missing values between the control and intervention arms and stratification by covariates of interest (age, sex, SES, education); 2) conducting Little’s MCAR test, as specified in our pre-analysis plan; 3) comparing the results of fully adjusted GEE models, which excluded participants with covariate missingness, to unadjusted GEE models, which did not exclude those participants; and 4) comparing GEE models to mixed effect models fit with full information maximum likelihood and robust to missingness under the MAR assumption.

Examination of the distribution of missing values between arms and stratification by covariates of interest revealed a similar pattern of missingness in both groups both overall and within covariate stratum. Little’s test was nonsignificant (p=0.45), indicating that the primary outcome was missing completely at random (MCAR) and suggesting a lack of bias from the exclusion of cases with missing outcome data. Further, comparing the results of fully adjusted GEE models to unadjusted GEE models showed no substantive difference in effect estimates. Similarly, comparing GEE models to mixed effect models fit with full information maximum likelihood and robust to missingness under the MAR assumption showed no substantive difference from the reported GEE results. Given the totality of these findings, we are confident that the exclusion of cases with missing data did not result in biased effect estimates.

Supplemental Tables

Supplemental Table 1: Types of repairs reported among households with a latrine at endline (n=272)

Repair type	Number of repairs	Repaired latrines (%)
**Door**	155	57.0%
**Roof**	0	0.0%
**Walls**	2	0.7%
**Slab cover**	40	14.7%
**Pan**	13	4.7%
**Pipe connection**	38	14.0%
**Pit lining**	52	19.1%
**Flooring**	57	21.0%
**Parapet**	1	0.4%
**Other**	8	2.9%

Note: Multiple repairs possible per household; n = 272 repairs among 268 households reporting having received repairs at endline. 382 Households were eligible for repairs, and 24 were lost to follow-up resulting in 75% (268) of 358 reporting having received repairs.

Supplemental Figure 2: **Baseline and endline values and difference in difference (DID) of latrine use at last defecation among individuals age five and older from latrine owning households, by study arm and sex.**

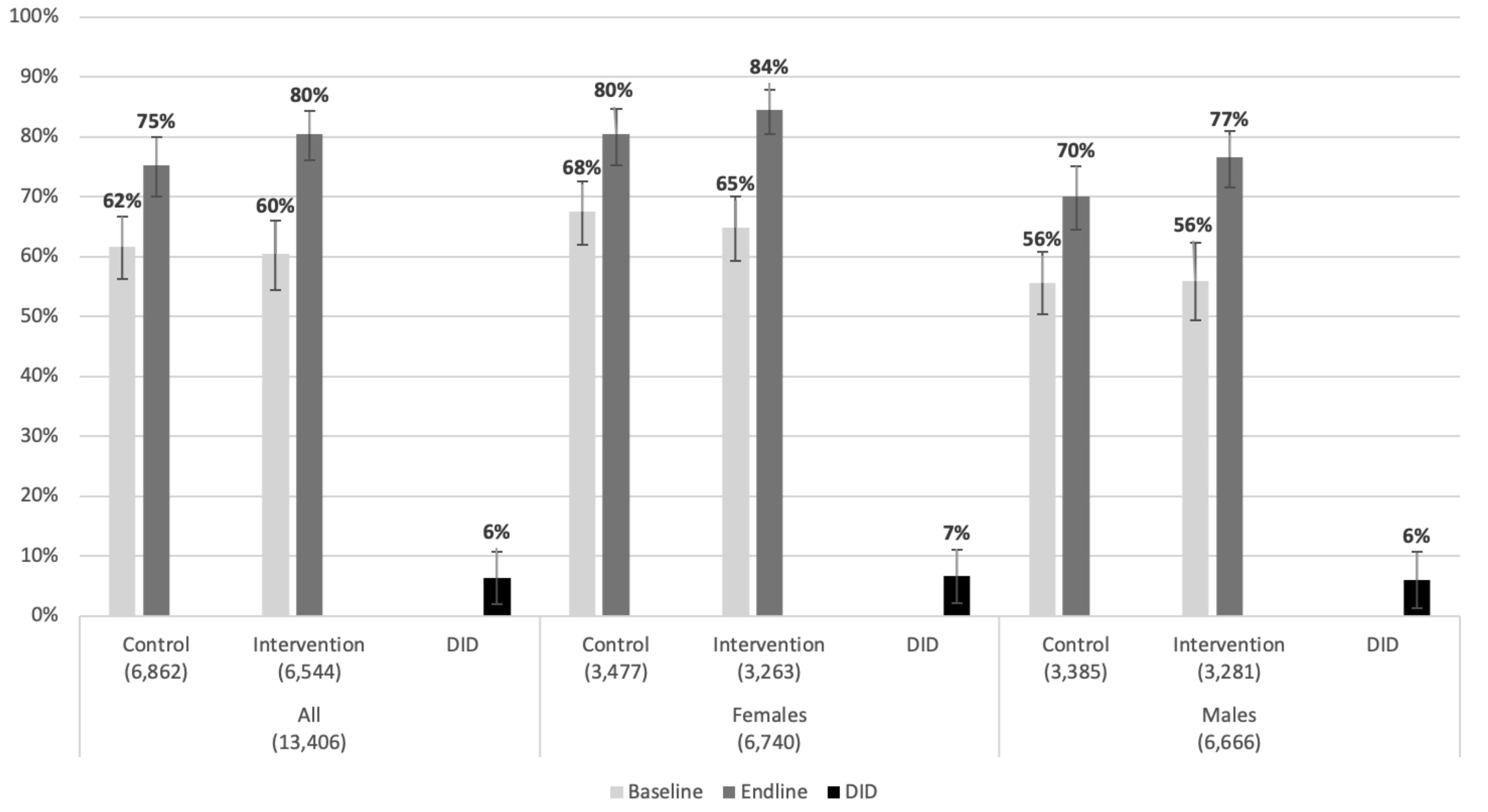

Supplemental Table 2: Baseline and Endline Latrine Use and Safe Child Feces Disposal, by Age Category

	N		Baseline Latrine Use and Safe Disposal of Child Feces, by Age Group[1]		Endline Latrine Use and Safe Disposal of Child Feces, by Age Group[1]	
	Intervention	Control	Intervention	Control	Intervention	Control
**Latrine Use**						
All age 5 and older	6544	6862	3954 (60.4%)	4231 (61.7%)	5267 (80.5%)	5170 (75.3%)
Age 5-12	719	726	456 (63.4%)	494 (68.0%)	612 (85.1%)	578 (79.6%)
Age 13-19	699	844	421 (60.2%)	557 (66.0%)	573 (82.0%)	646 (76.5%)
Age 20-29	1171	1216	786 (67.1%)	810 (66.6%)	988 (84.4%)	979 (80.5%)
Age 30-39	1061	992	706 (66.5%)	653 (65.8%)	889 (83.8%)	768 (77.4%)
Age 40-49	983	1043	554 (56.4%)	616 (59.1%)	769 (78.2%)	782 (75.0%)
Age 50-59	835	901	466 (55.8%)	512 (56.8%)	659 (78.9%)	659 (73.1%)
Age 60+	1076	1140	565 (52.5%)	589 (51.7%)	777 (72.2%)	758 (66.5%)
**JMP Defined Safe Child Feces Disposal**						
All children under age 5	377	397	87 (23.1%)	86 (21.7%)	226 (60%)	172 (43.3%)
0-12 Months	71	66	7 (9.9%)	3 (4.6%)	30 (42.3%)	8 (12.2%)
13-23 Months	59	62	2 (3.4%)	3 (4.8%)	21 (35.6%)	15 (24.2%)
24-35 Months	87	87	12 (13.8%)	11 (12.6%)	56 (64.4%)	35 (40.2%)
36-47 Months	79	90	26 (32.9%)	29 (32.2%)	55 (69.6%)	54 (60.0%)
48-59 Months	81	92	40 (49.4%)	40 (43.5%)	64 (79.1%)	60 (65.2%)
**Latrine Use Among Children Under Age 5**						
All children under age 5	376	396	58 (15.4%)	72 (18.2%)	157 (41.8%)	148 (37.4%)
0-12 Months	71	66	0 (0.0%)	1 (1.5%)	1 (1.4%)	2 (3.0%)
13-23 Months	58	62	0 (0.0%)	0 (0.0%)	6 (10.3%)	11 (17.7%)
24-35 Months	87	87	5 (5.8%)	6 (6.9%)	40 (46.0%)	26 (29.9%)
36-47 Months	79	89	22 (27.9%)	27 (30.3%)	47 (59.5%)	50 (56.2%)
48-59 Months	81	82	31 (38.3%)	38 (41.3%)	63 (77.8%)	59 (64.1%)
**Caregiver Safe Child Feces Disposal**						
All children under age 5	199	207	12 (6.0%)	7 (3.4%)	67 (33.7%)	22 (10.6%)
0-12 Months	70	63	7 (10.0%)	2 (3.2%)	29 (41.4%)	6 (9.5%)
13-23 Months	52	51	2 (3.9%)	1 (2.0%)	15 (28.9%)	4 (7.8%)
24-35 Months	47	59	1 (2.1%)	4 (6.8%)	16 (34.0%)	9 (15.3%)
36-47 Months	23	27	1 (4.4%)	0 (0.0%)	6 (26.1%)	3 (11.1%)
48-59 Months	7	7	1 (14.3%)	0 (0.0%)	1 (14.3%)	0 (0.0%)

1. Baseline and endline proportions are unadjusted.

Supplemental Table 3: Effect of intervention on latrine use among individuals age five and older (n=13406)

	Effect size	SE	95% CI%	p-value
DID (Intervention*Time)	0.064	0.022	0.020; 0.107	<0.01
Intervention	-0.014	0.033	-0.079; 0.050	0.67
Time	0.137	0.013	0.111; 0.162	<0.0001
Number of HH members	-0.007	0.003	-0.013; 0.001	0.03
SES				
Quintile 1	*Ref*			
Quintile 2	0.086	0.023	0.040; 0.132	<0.0001
Quintile 3	0.153	0.021	0.111; 0.195	<0.0001
Quintile 4	0.239	0.019	0.202; 0.275	<0.0001
Quintile 5	0.326	0.022	0.282; 0.370	<0.0001
Sex				
Male	-0.093	0.007	-0.106; -0.080	<0.0001
Female	*Ref*			
Education of male HH head (grade)	0.010	0.001	0.008; 0.013	<0.0001
Education of female HH head	0.004	0.001	0.001; 0.007	<0.01
Age category				
5–12	0.041	0.011	0.019; 0.063	<0.001
13–19	0.038	0.012	0.015; 0.061	<0.01
20–29	0.044	0.011	0.023; 0.065	<0.0001
30–39	*Ref*			
40–49	-0.027	0.012	-0.050; 0.003	0.03
50–59	-0.046	0.013	-0.071; -0.020	<0.01
60+	-0.090	0.012	-0.114; -0.065	<0.0001
Intercept	0.446	0.038	0.371; 0.521	<0.0001

Notes: DID = difference in difference, SE = standard error; HH = household. Adjusted for clustering, ITT analysis

Supplementary Table 4: Effect of intervention on latrine use among females age five and older (n=6740)

	Effect size	SE	95% CI%	p-value
DID (Intervention*Time)	0.066	0.023	0.022; 0.111	<0.01
Intervention	-0.025	0.031	-0.087; 0.036	0.42
Time	0.129	0.013	0.103; 0.155	<0.0001
Number of HH members	-0.007	0.003	-0.013; -0.001	0.03
SES				
Quintile 1	*Ref*			
Quintile 2	0.094	0.026	0.044; 0.144	<0.001
Quintile 3	0.168	0.022	0.125; 0.212	<0.0001
Quintile 4	0.243	0.022	0.201; 0.286	<0.0001
Quintile 5	0.322	0.024	0.275; 0.369	<0.0001
Education of male HH head	0.009	0.002	0.006; 0.012	<0.0001
Education of female HH head	0.004	0.002	0.000; 0.007	0.03
Age category				
5–12	0.022	0.016	-0.009; 0.053	0.16
13–19	0.059	0.014	0.031; 0.088	<0.0001
20–29	0.059	0.012	0.036; 0.083	<0.0001
30–39	*Ref*			
40–49	-0.008	0.015	-0.037; 0.020	0.57
50–59	-0.047	0.016	-0.078; -0.016	<0.01
60+	-0.077	0.015	-0.107; -0.048	<0.0001
Intercept	0.451	0.041	0.371; 0.531	<0.0001

Notes: DID = difference in difference, SE = standard error; HH = household. Estimates adjusted for clustering, ITT analysis.

Supplementary Table 5: Effect of intervention on latrine use among males age five and older (n=6666)

	Effect size	SE	95% CI%	p-value
DID (Intervention*Time)	0.061	0.024	0.014; 0.108	0.01
Intervention	0.004	0.036	-0.074; 0.067	0.92
Time	0.144	0.014	0.117; 0.172	<0.0001
Number of HH members	-0.008	0.004	-0.015; 0.000	0.04
SES				
Quintile 1	*Ref*			
Quintile 2	0.078	0.026	0.028; 0.128	<0.01
Quintile 3	0.139	0.024	0.091; 0.187	<0.0001
Quintile 4	0.235	0.020	0.196; 0.274	<0.0001
Quintile 5	0.330	0.026	0.280; 0.380	<0.0001
Education of male HH head	0.011	0.001	0.008; 0.014	<0.0001
Education of female HH head	0.005	0.002	0.002; 0.008	<0.01
Age category				
5–12	0.054	0.016	0.023; 0.086	0.0008
13–19	0.016	0.017	-0.019; 0.050	0.37
20–29	0.027	0.015	-0.002; 0.057	0.07
30–39	*Ref*			
40–49	-0.049	0.016	-0.080; -0.018	<0.01
50–59	-0.046	0.017	-0.080; -0.013	<0.01
60+	-0.102	0.016	-0.132; -0.071	<0.0001
Intercept	0.352	0.040	0.274; 0.431	<0.0001

Notes: DID = difference in difference, SE = standard error; HH = household. Estimates adjusted for clustering, ITT analysis.

Supplementary Table 6: Effect of intervention on proportion of latrines reported by enumerators to be in use (N=3281)

	**Effect size**	**SE**	**95% CI%**	**p-value**
DID (Intervention*Time)	0.043	0.019	0.006; 0.080	0.02
Intervention	-0.007	0.029	-0.065; 0.050	0.80
Time	0.076	0.011	0.054; 0.099	<0.0001
Number of HH members	0.005	0.003	-0.001; 0.011	0.09
SES				
Quintile 1	*Ref*			
Quintile 2	0.086	0.022	0.042; 0.130	<0.001
Quintile 3	0.163	0.027	0.110; 0.216	<0.0001
Quintile 4	0.238	0.023	0.193; 0.282	<0.0001
Quintile 5	0.302	0.023	0.256; 0.348	<0.0001
Education of male HH head	0.005	0.002	0.002; 0.009	<0.01
Education of female HH head	0.001	0.001	-0.002; 0.004	0.44
Intercept	0.504	0.033	0.439; 0.569	<0.0001

Notes: DID = difference in difference, SE = standard error; HH = household. Estimates adjusted for clustering, ITT analysis.

Supplemental Table 7: Effect of intervention on latrine use among individuals age five and older, adjusting for participation in nested measurement survey (repeated measures) (n=13406)

	**Effect size**	**SE**	**95% CI%**	**p-value**
DID (Intervention*Time)	0.066	0.023	0.022; 0.111	<0.01
Intervention	-0.018	0.034	-0.085; 0.049	0.60
Time	0.137	0.013	0.111; 0.163	<0.001
Number of HH members	-0.007	0.003	-0.013; 0.001	0.03
SES				
Quintile 1	*Ref*			
Quintile 2	0.083	0.024	0.040; 0.130	<0.001
Quintile 3	0.150	0.021	0.107; 0.191	<0.001
Quintile 4	0.235	0.018	0.199; 0.271	<0.001
Quintile 5	0.320	0.022	0.277; 0.364	<0.001
Sex				
Male	-0.092	0.007	-0.104; -0.080	<0.001
Female	*Ref*			
Education of male HH head (grade)	0.011	0.001	0.008; 0.013	<0.001
Education of female HH head	0.004	0.001	0.001; 0.007	<0.01
Age category				
5–12	0.039	0.011	0.017; 0.061	<0.001
13–19	0.038	0.011	0.015; 0.061	0.001
20–29	0.043	0.010	0.023; 0.063	<0.001
30–39	*Ref*			
40–49	-0.028	0.011	-0.050; 0.005	0.02
50–59	-0.045	0.013	-0.070; -0.020	<0.001
60+	-0.090	0.012	-0.114; -0.065	<0.001
Intercept	0.445	0.038	0.370; 0.520	<0.001

Notes: DID = difference in difference, SE = standard error; HH = household. Adjusted for clustering, ITT analysis

Supplementary Table 8: Effect of intervention on latrine use and safe child feces disposal (unadjusted)

	Effect size	SE	95% CI%	p-value
**Latrine use among persons aged 5 and older (N=15781)**				
DID (Intervention*Time)	0.075	0.024	0.028; 0.121	<0.01
Intervention	-0.008	0.040	-0.086; 0.071	0.85
Time	0.133	0.013	0.106; 0.159	<0.0001
Intercept	0.620	0.027	0.567; 0.674	<0.0001
**JMP Defined Safe Child Faeces Disposal (N=979)**				
DID (Intervention*Time)	0.143	0.037	0.071; 0.215	<0.0001
Intervention	0.025	0.036	-0.046; 0.095	0.49
Time	0.210	0.024	0.164; 0.256	<0.0001
Intercept	0.216	0.026	0.165; 0.267	<0.0001
**Latrine Use Among Children Under Age 5 (N=981)**				
DID (Intervention*Time)	0.063	0.033	-0.002; 0.127	0.06
Intervention	-0.024	0.030	-0.083; 0.036	0.43
Time	0.190	0.023	0.146; 0.234	<0.0001
Intercept	0.183	0.023	0.137; 0.229	<0.0001
**Caregiver Safe Child Faeces Disposal** (N=974)				
DID (Intervention*Time)	0.148	0.041	0.067; 0.230	<0.001
Intervention	0.031	0.034	-0.036; 0.098	0.37
Time	0.134	0.029	0.077; 0.191	<0.0001
Intercept	0.203	0.023	0.157; 0.249	<0.0001

Supplementary Table 9: Effect of Intervention on JMP defined safe child feces disposal (n=774)

	Effect size	SE	95% CI%	p-value
DID (Intervention*Time)	0.152	0.037	0.795; 0.225	<0.0001
Intervention	0.010	0.035	-0.059; 0.079	0.78
Time	0.217	0.025	0.168; 0.265	<0.0001
Number of HH members	-0.003	0.007	-0.016; 0.010	0.68
SES				
Quintile 1	*Ref*			
Quintile 2	-0.015	0.056	-0.125; 0.094	0.79
Quintile 3	0.018	0.042	-0.063; 0.100	0.66
Quintile 4	0.092	0.048	-0.001; 0.186	0.05
Quintile 5	0.148	0.046	0.589; 0.237	<0.01
Sex				
Male	-0.036	0.025	-0.084; 0.012	0.14
Female	*Ref*			
Education of male HH head (grade)	0.007	0.003	0.000; 0.014	0.04
Education of female HH head	0.005	0.004	-0.003; 0.014	0.23
Intercept	0.099	0.068	-0.035; 0.232	0.15

Notes: DID = difference in difference, SE = standard error; HH = household. Estimates adjusted for clustering, ITT analysis.

Supplementary Table 10: Effect of Intervention on JMP defined safe child feces disposal, female children (n=403)

	Effect size	SE	95% CI%	p-value
DID (Intervention*Time)	0.104	0.052	0.002; 0.205	0.05
Intervention	0.000	0.043	-0.084; 0.085	0.99
Time	0.245	0.034	0.180; 0.311	<0.0001
Number of HH members	0.003	0.009	-0.015; 0.021	0.76
SES				
Quintile 1	*Ref*			
Quintile 2	-0.044	0.073	-0.188; 0.099	0.55
Quintile 3	0.028	0.057	-0.083; 0.139	0.62
Quintile 4	0.122	0.058	0.009; 0.235	0.04
Quintile 5	0.137	0.066	0.007; 0.267	0.04
Education of male HH head (grade)	0.003	0.005	-0.008; 0.013	0.65
Education of female HH head	0.007	0.005	-0.004; 0.018	0.21
Intercept	0.091	0.089	-0.083; 0.265	0.31

Notes: DID = difference in difference, SE = standard error; HH = household. Estimates adjusted for clustering, ITT analysis.

Supplementary Table 11: Effect of Intervention on JMP defined safe child feces disposal, male children (n=371)

	Effect size	SE	95% CI%	p-value
DID (Intervention*Time)	0.205	0.047	0.113; 0.297	<0.0001
Intervention	0.014	0.049	-0.083; 0.110	0.78
Time	0.185	0.036	0.114; 0.256	<0.0001
Number of HH members	-0.010	0.010	-0.030; 0.009	0.30
SES				
Quintile 1	*Ref*			
Quintile 2	0.021	0.079	-0.135; 0.177	0.79
Quintile 3	-0.002	0.064	-0.127; 0.123	0.98
Quintile 4	0.068	0.077	-0.083; 0.220	0.38
Quintile 5	0.174	0.067	0.042; 0.306	<0.01
Education of male HH head (grade)	0.012	0.004	0.003; 0.021	<0.01
Education of female HH head	0.003	0.005	-0.007; 0.013	0.55
Intercept	0.083	0.095	-0.104; 0.269	0.39

Notes: DID = difference in difference, SE = standard error; HH = household. Estimates adjusted for clustering, ITT analysis.

<u>Supplemental</u> Figure 3: Baseline and endline values and difference in difference (DID) of JMP-defined safe child faeces disposal, child latrine use, and caregiver safe disposal of child faeces among children under age five and older from latrine owning households, by study arm and sex

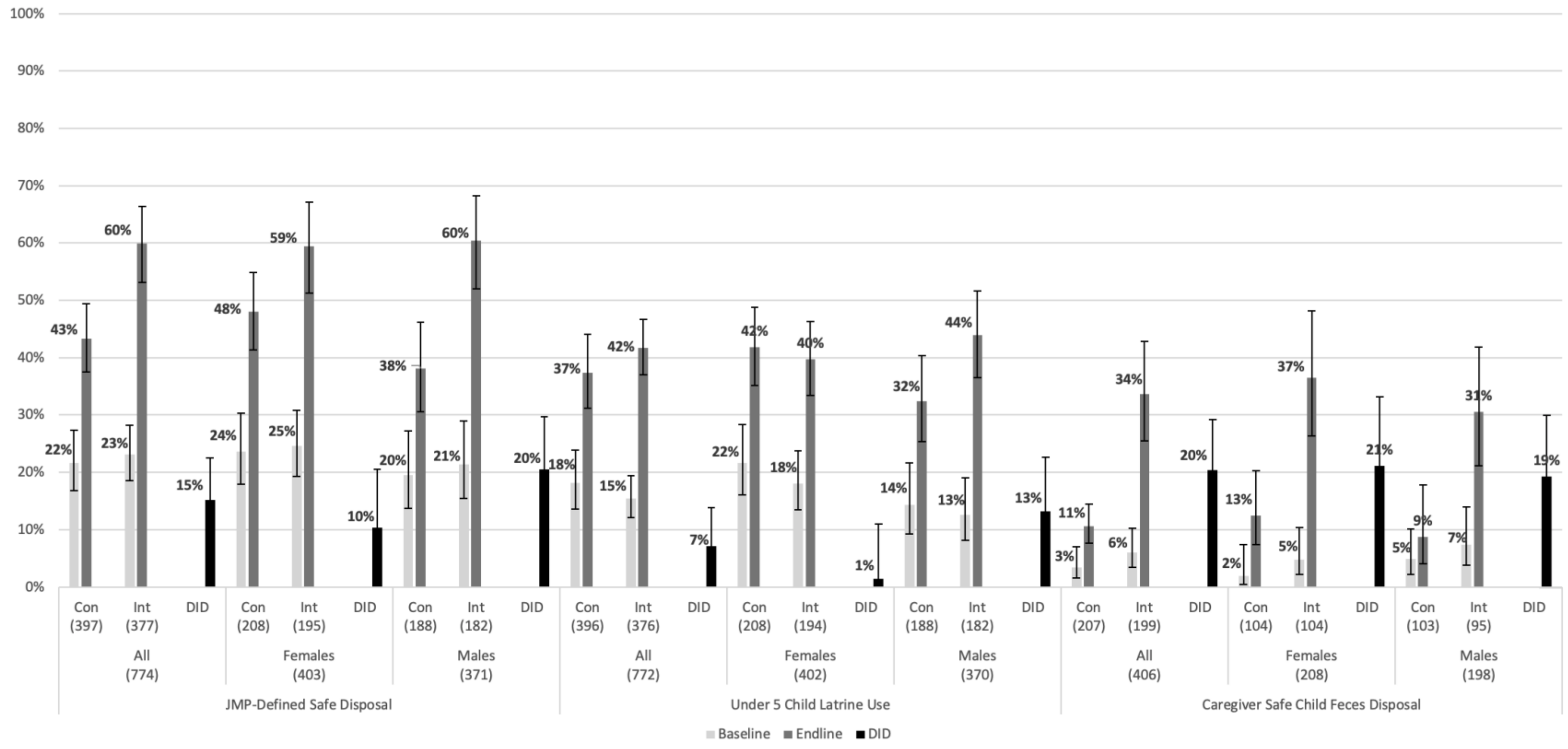

Supplementary Table 12: Effect of intervention on latrine use among children under age five (n=772)

	Effect size	SE	95% CI%	p-value
DID (Intervention*Time)	0.071	0.035	0.004; 0.139	0.04
Intervention	-0.029	0.031	-0.090; 0.031	0.34
Time	0.192	0.025	0.143; 0.241	<0.0001
Number of HH members	0.001	0.007	-0.012; 0.014	0.88
SES				
Quintile 1	*Ref*			
Quintile 2	-0.052	0.047	-0.143; 0.040	0.27
Quintile 3	-0.002	0.039	-0.079; 0.074	0.95
Quintile 4	0.034	0.045	-0.054; 0.123	0.45
Quintile 5	0.076	0.044	-0.011; 0.163	0.09
Sex				
Male	-0.042	0.023	-0.087; 0.003	0.07
Female	*Ref*			
Education of male HH head (grade)	0.004	0.004	-0.003; 0.011	0.24
Education of female HH head	0.010	0.004	0.003; 0.017	0.01
Intercept	0.087	0.069	-0.048; 0.222	0.21

Notes: DID = difference in difference, SE = standard error; HH = household. Estimates adjusted for clustering, ITT analysis.

Supplementary Table 13: Effect of intervention on latrine use among children under age, female children (n=402)

	Effect size	SE	95% CI%	p-value
DID (Intervention*Time)	0.015	0.048	-0.080; 0.109	0.76
Intervention	-0.039	0.041	-0.119; 0.041	0.34
Time	0.202	0.029	0.144; 0.260	<0.0001
Number of HH members	0.007	0.009	-0.11; 0.025	0.44
SES				
Quintile 1	*Ref*			
Quintile 2	-0.053	0.063	-0.011; 0.069	0.39
Quintile 3	0.027	0.054	-0.079; 0.133	0.62
Quintile 4	0.076	0.054	-0.030; 0.181	0.16
Quintile 5	0.081	0.064	-0.045; 0.208	0.21
Education of male HH head (grade)	0.000	0.005	-0.010; 0.010	0.96
Education of female HH head	0.010	0.005	0.000; 0.021	0.05
Intercept	0.070	0.090	-0.106; 0.245	0.44

Notes: DID = difference in difference, SE = standard error; HH = household. Estimates adjusted for clustering, ITT analysis.

Supplementary Table 14: Effect of intervention on latrine use among children under age, male children (n=370)

	Effect size	SE	95% CI%	p-value
DID (Intervention*Time)	0.132	0.048	0.038; 0.227	<0.01
Intervention	-0.029	0.043	-0.112; 0.055	0.50
Time	0.181	0.035	0.112; 0.250	<0.0001
Number of HH members	-0.008	0.010	-0.027; 0.012	0.44
SES				
Quintile 1	*Ref*			
Quintile 2	-0.042	0.067	-0.173; 0.088	0.53
Quintile 3	-0.033	0.062	-0.154; 0.089	0.60
Quintile 4	-0.001	0.070	-0.140; 0.137	0.99
Quintile 5	0.087	0.065	-0.039; 0.214	0.18
Education of male HH head (grade)	0.009	0.005	0.000; 0.018	0.04
Education of female HH head	0.007	0.004	-0.001; 0.016	0.08
Intercept	0.076	0.090	-0.100; 0.252	0.04

Notes: DID = difference in difference, SE = standard error; HH = household. Estimates adjusted for clustering, ITT analysis.

Supplementary Table 15: Effect of Intervention on caregiver safe disposal of child faeces (n=406)

	Effect size	SE	95% CI%	p-value
DID (Intervention*Time)	0.204	0.045	0.116; 0.292	<0.0001
Intervention	0.026	0.021	-0.016; 0.067	0.23
Time	0.072	0.018	0.038; 0.107	<0.0001
Number of HH members	-0.012	0.006	-0.023; 0.000	0.05
SES				
Quintile 1	*Ref*			
Quintile 2	0.075	0.041	-0.005; 0.155	0.07
Quintile 3	0.068	0.035	-0.001; 0.137	0.05
Quintile 4	0.119	0.037	0.046; 0.192	<0.01
Quintile 5	0.172	0.039	0.095; 0.249	<0.0001
Sex				
Male	-0.008	0.024	-0.055; 0.038	0.73
Female	*Ref*			
Education of male HH head (grade)	0.003	0.003	-0.003; 0.010	0.34
Education of female HH head	-0.006	0.004	-0.013; 0.001	0.11
Intercept	0.013	0.041	-0.068; 0.095	0.75

Notes: DID = difference in difference, SE = standard error; HH = household. Estimates adjusted for clustering, ITT analysis.

Supplementary Table 16: Effect of Intervention on caregiver safe disposal of child faeces, female children (n= 208)

	Effect size	SE	95% CI%	p-value
DID (Intervention*Time)	0.212	0.062	0.091; 0.332	<0.001
Intervention	0.011	0.027	-0.043; 0.065	0.68
Time	0.106	0.031	0.044; 0.167	<0.001
Number of HH members	-0.007	0.008	-0.023; 0.008	0.36
SES				
Quintile 1	*Ref*			
Quintile 2	0.063	0.055	-0.045; 0.172	0.25
Quintile 3	0.060	0.046	-0.031; 0.150	0.20
Quintile 4	0.131	0.051	0.031; 0.232	0.01
Quintile 5	0.147	0.047	0.054; 0.240	<0.01
Education of male HH head (grade)	0.000	0.005	-0.009; 0.009	0.96
Education of female HH head	-0.004	0.004	-0.012; 0.005	0.39
Intercept	-0.007	0.059	-0.122; 0.108	0.90

Notes: DID = difference in difference, SE = standard error; HH = household. Estimates adjusted for clustering, ITT analysis.

Supplementary Table 17: Effect of Intervention on caregiver safe disposal of child faeces, male children (n=198)

	Effect size	SE	95% CI%	p-value
DID (Intervention*Time)	0.193	0.055	0.085; 0.300	0.0004
Intervention	0.045	0.030	-0.015; 0.105	0.14
Time	0.039	0.025	-0.010; 0.088	0.12
Number of HH members	-0.016	0.009	-0.033; 0.002	0.08
SES				
Quintile 1	*Ref*			
Quintile 2	0.085	0.058	-0.028; 0.197	0.14
Quintile 3	0.078	0.052	-0.023; 0.179	0.13
Quintile 4	0.115	0.051	0.015; 0.214	0.02
Quintile 5	0.204	0.062	0.083; 0.325	<0.01
Education of male HH head (grade)	0.006	0.005	-0.004; 0.016	0.21
Education of female HH head	-0.008	0.006	-0.019; 0.004	0.19
Intercept	0.015	0.063	-0.109; 0.138	0.82

Notes: DID = difference in difference, SE = standard error; HH = household. Estimates adjusted for clustering, ITT analysis
